# Phosphoglycolate salvage in a chemolithoautotroph using the Calvin cycle

**DOI:** 10.1073/pnas.2012288117

**Published:** 2020-08-20

**Authors:** Nico J. Claassens, Giovanni Scarinci, Axel Fischer, Avi I. Flamholz, William Newell, Stefan Frielingsdorf, Oliver Lenz, Arren Bar-Even

**Affiliations:** ^a^Systems and Synthetic Metabolism Lab, Max Planck Institute of Molecular Plant Physiology, 14476 Potsdam-Golm, Germany;; ^b^Department of Molecular and Cell Biology, University of California, Berkeley, CA 94720;; ^c^Institut für Chemie, Physikalische Chemie, Technische Universität Berlin, 10623 Berlin, Germany

**Keywords:** CO_2_ fixation, hydrogen-oxidizing bacteria, glycolate oxidation, malate synthase, glycolate secretion

## Abstract

Carbon fixation via the Calvin cycle is constrained by the side activity of Rubisco with dioxygen, generating 2-phosphoglycolate. The metabolic recycling of phosphoglycolate was extensively studied in photoautotrophic organisms, including plants, algae, and cyanobacteria, where it is referred to as photorespiration. While receiving little attention so far, aerobic chemolithoautotrophic bacteria that operate the Calvin cycle independent of light must also recycle phosphoglycolate. As the term photorespiration is inappropriate for describing phosphoglycolate recycling in these nonphotosynthetic autotrophs, we suggest the more general term “phosphoglycolate salvage.” Here, we study phosphoglycolate salvage in the model chemolithoautotroph *Cupriavidus necator* H16 (*Ralstonia eutropha* H16) by characterizing the proxy process of glycolate metabolism, performing comparative transcriptomics of autotrophic growth under low and high CO_2_ concentrations, and testing autotrophic growth phenotypes of gene deletion strains at ambient CO_2_. We find that the canonical plant-like C_2_ cycle does not operate in this bacterium, and instead, the bacterial-like glycerate pathway is the main route for phosphoglycolate salvage. Upon disruption of the glycerate pathway, we find that an oxidative pathway, which we term the malate cycle, supports phosphoglycolate salvage. In this cycle, glyoxylate is condensed with acetyl coenzyme A (acetyl-CoA) to give malate, which undergoes two oxidative decarboxylation steps to regenerate acetyl-CoA. When both pathways are disrupted, autotrophic growth is abolished at ambient CO_2_. We present bioinformatic data suggesting that the malate cycle may support phosphoglycolate salvage in diverse chemolithoautotrophic bacteria. This study thus demonstrates a so far unknown phosphoglycolate salvage pathway, highlighting important diversity in microbial carbon fixation metabolism.

## Significance

The Calvin cycle is the most important carbon fixation pathway in the biosphere. However, its carboxylating enzyme Rubisco also accepts oxygen, thus producing 2-phosphoglycolate. Phosphoglycolate salvage pathways were extensively studied in photoautotrophs but remain uncharacterized in chemolithoautotrophs using the Calvin cycle. Here, we study phosphoglycolate salvage in the chemolithoautotrophic model bacterium *Cupriavidus necator* H16. We demonstrate that this bacterium mainly reassimilates 2-phosphoglycolate via the glycerate pathway. Upon disruption of this pathway, a secondary route, which we term the malate cycle, supports photorespiration by completely oxidizing 2-phosphoglycolate to CO_2_. While the malate cycle was not previously known to metabolize 2-phosphoglycolate in nature, a bioinformatic analysis suggests that it may support phosphoglycolate salvage in diverse chemoautotrophic bacteria.

**Fig. 1.**
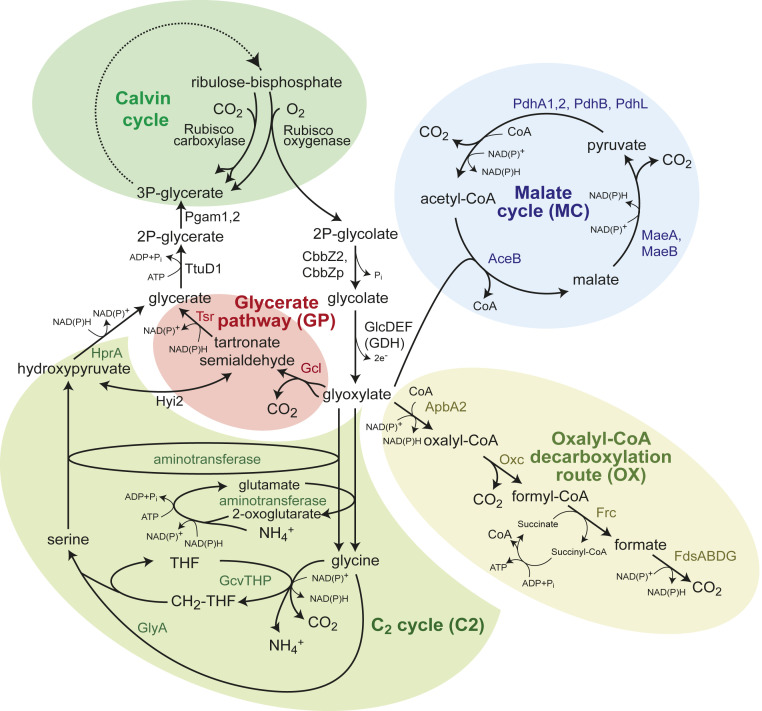
Candidate pathways supporting phosphoglycolate salvage in *C. necator*; 2PG is first dephosphorylated to glycolate and then oxidized, by the glycolate dehydrogenase complex, to give glyoxylate. Glyoxylate can be further metabolized via four routes: the C_2_ cycle (i.e., the plant phosphoglycolate salvage pathway), the glycerate pathway, the oxalyl-CoA decarboxylation pathway, and malate cycle. The latter two routes completely oxidize glycoxylate to CO_2_. Note that that malate cycle may also proceed via oxidation of malate to oxaloacetate, which can be converted to pyruvate either directly or via phosphoenolpyruvate. AceB, malate synthase; ApbA2, CoA-acylating glyoxylate dehydrogenase; CbbZ2, CbbZp, 2-phosphoglycolate phosphatase; FdsABDG, formate dehydrogenase complex; Frc, formyl-CoA transferase; Gcl, glyoxylate carboligase; GcvTHP, glycine cleavage systems; GlcDEF, glycolate dehydrogenase complex; GlyA, serine hydroxymethyltransferase; HprA, hydroxypyruvate reductase; Hyi2, hydroxypyruvate isomerase; MaeA, malic enzyme A; MaeB, malic enzyme B; Oxc, oxalyl-CoA decarboxylase; PdhA1,2, PdhB, PdhL, pyruvate dehydrogenase complex; Pgam1,2, phosphoglycerate mutatase; Rubisco, Ribulose-bisphosphate carboxylase/oxygenase; THF, tetrahydrofolate; Tsr, tartronate semialdehyde reductase; TtuD1, glycerate kinase.

The Calvin cycle is responsible for the vast majority of carbon fixation in the biosphere. Among several other factors, its activity is constrained by the low rate and the limited substrate specificity of its carboxylating enzyme Rubisco. The oxygenase side activity of Rubisco converts ribulose 1,5-bisphosphate into 3-phosphoglycerate (3PG) and 2-phosphoglycolate (2PG), the latter of which represents a loss of carbon from the Calvin cycle and can inhibit its enzymes (1, 2). The metabolic recycling of 2PG, usually termed “photorespiration,” is an essential process for organisms that grow autotrophically via the Calvin cycle (3). Photorespiration has been extensively studied in photosynthetic organisms, including plants, algae, and cyanobacteria (4–7). The only identified photorespiration pathway in plants is the so-called C_2_ cycle (Fig. 1), in which 2PG is first dephosphorylated to glycolate, then oxidized to glyoxylate, and subsequently aminated to glycine. One glycine molecule is decarboxylated to give methylenetetrahydrofolate (methylene-THF), which reacts with another glycine to yield serine. Serine is then deaminated to hydroxypyruvate, further reduced to glycerate, and finally phosphorylated to generate the Calvin cycle intermediate 3PG.

In the cyanobacterium *Synechocystis* sp. PCC6803, gene deletion studies were used to demonstrate the activity of two photorespiratory routes in addition to the C_2_ cycle (5, 8). In the glycerate pathway, two glyoxylate molecules are condensed to tartronate semialdehyde, which is subsequently reduced to glycerate and phosphorylated to 3PG (Fig. 1). Alternatively, in the oxalate decarboxylation pathway, glyoxylate is oxidized to oxalate and decarboxylated to formate, which is finally oxidized to CO_2_ (Fig. 1). Growth at ambient CO_2_ was abolished only when all three pathways were deleted, indicating that each of these routes can participate in photorespiration (5).

The term photorespiration was initially coined to describe the light-dependent O_2_ consumption and CO_2_ release in plant leaves (9–12). Even though the name implies otherwise, photorespiration-like metabolism is not restricted to photoautotrophs. In fact, a wide range of chemolithoautotrophic microorganisms employs the Calvin cycle, including autotrophic bacteria that oxidize dihydrogen, ferrous iron, sulfur, or ammonia (13, 14). Those chemolithoautotrophs that employ the Calvin cycle under aerobic conditions must also cope with the oxygenase side activity of Rubisco by recycling or removing 2PG. Yet, despite the obvious physiological significance of photorespiration-like metabolism in chemolithoautotrophs, it has so far received only scarce attention.

Since the term photorespiration is ill-suited to describe the recycling of 2PG in light-independent autotrophs, we suggest using the more general term “Rubisco-related 2-phosphoglycolate salvage” or for short, “phosphoglycolate salvage.” A similar term was used before in a few publications when referring to photorespiration (15–17). Importantly, “salvage” can refer to reassimilation of 2PG to central metabolism, as by the C_2_ cycle and the glycerate pathway, or to the complete oxidation of 2PG, as in the oxalate decarboxylation pathway, in which case the term refers to the recycling of reducing power and CO_2_, which can reenter the Calvin cycle.

In this study, we explore metabolic routes involved in phosphoglycolate salvage in *Cupriavidus necator* H16 (formerly known as *Ralstonia eutropha* H16 or *Alcaligenes eutrophus* H16), the best-studied chemolithoautotrophic microorganism that uses the Calvin cycle under aerobic conditions (but also under anaerobic conditions with nitrate as electron acceptor) (18–20). Unlike cyanobacteria, *C. necator* does not harbor a CO_2_ concentrating mechanism (i.e., a carboxysome with appropriate inorganic carbon transporters), as evident from the relatively high CO_2_ specificity of its Rubisco, which falls within the range reported for plants but is much higher than that found in cyanobacteria (21–23). Very little is known about phosphoglycolate salvage in *C. necator*. Previous studies found evidence only for the first steps of 2PG metabolism (i.e., its dephosphorylation followed by glycolate oxidation) (24–26). However, the specific routes that metabolize glyoxylate remain elusive.

We identify the native phosphoglycolate salvage pathways of *C. necator* by performing comparative transcriptomic analysis and conducting growth experiments with gene deletion strains. We show that the C_2_ cycle and decarboxylation via oxalate do not support phosphoglycolate salvage in *C. necator*. We find that the glycerate pathway is the primary phosphoglycolate salvage route in this bacterium. A second pathway, which we term the malate cycle, carries all phosphoglycolate salvage flux when the glycerate pathway is deleted. This route was previously unknown to operate in nature and can completely oxidize glyoxylate to CO_2_. In this cycle, glyoxylate is condensed with acetyl-CoA (coenzyme A) to generate malate, which then undergoes oxidative decarboxylation twice to regenerate acetyl-CoA (Fig. 1). Only when both the glycerate pathway and the malate cycle are deleted is autotrophic growth at ambient CO_2_ abolished. This study therefore fills an important gap in our understanding of chemolithoautotrophic metabolism.

## Results

### Multiple Pathways in *C. necator* Support Growth on Glycolate.

**Fig. 2.**
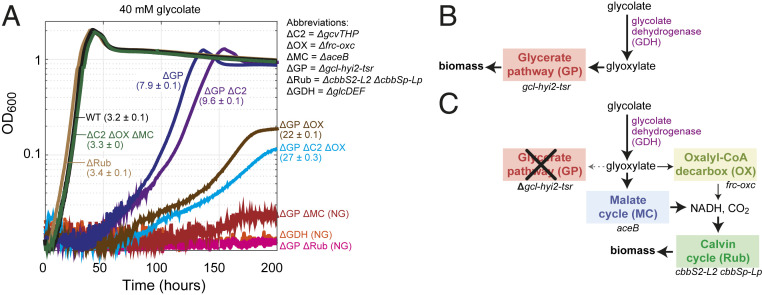
Growth of *C. necator* gene deletion strains on glycolate. (*A*) Growth experiments were conducted in 96-well plate readers in minimal medium (JMM) supplemented with 40 mM sodium glycolate. Doubling times (hours) and SDs of biological triplicates are shown in parentheses. NG corresponds to no growth. Triplicate growth experiments showed identical growth curves (±5%); hence, representative curves are shown. (*B*) The main pathway for growth on glycolate in *C. necator* proceeds via glycolate dehydrogenase and the glycerate pathway, which assimilated glyoxylate into central metabolism. (*C*) In the absence of the glycerate pathway, glyoxylate is decarboxylated mostly via the malate cycle and partly via oxalyl-CoA. Generated CO_2_ and reducing equivalents are utilized in the Calvin cycle to support biomass formation. ΔC2, C_2_ cycle knockout (*ΔgcvTHP*); ΔGDH, glycolate dehydrogenase knockout (*ΔglcD-kch-glcE-glcF*); ΔGP, glycerate pathway knockout (*Δgcl-hyi-tsr*); ΔMC, malate cycle knockout (*ΔaceB*); ΔOX, oxalate decarboxylation knockout (*frc-oxc*); ΔRub, Rubisco knockout (*ΔcbbS2-cbbL2 ΔcbbSp− ΔcbbLp*); WT, wild type.

As glycolate metabolism is at the core of phosphoglycolate salvage (Fig. 1), we decided to start by exploring the metabolic pathways that can support the growth of *C. necator* on this C_2_ carbon source. First, we focused on the initial oxidation of glycolate to glyoxylate, which is the first step in all glycolate-metabolizing routes. The operon *glcDEF* is annotated to encode different subunits of the glycolate dehydrogenase complex (27). The gene *kch*, which is also part of the operon, is annotated as an ion transport channel, but as it shows homology to flavin adenine dinucleotide (FAD)-binding proteins, including subunit *D* of glycolate dehydrogenase from *Ralstonia syzygii* R24, it is more likely to encode another subunit of the complex. While a wild-type *C. necator* could efficiently grow on glycolate (doubling time of 3.2 ± 0.1 h) (WT in Fig. 2*A*), a strain deleted in the *glcDEF* operon failed to grow on this carbon source (ΔGDH in Fig. 2*A*). This confirms that glycolate dehydrogenase complex plays an essential role in oxidizing glycolate to glyoxylate.

Next, we explored possible routes for glyoxylate metabolism. *C. necator* harbors genes encoding all of the enzymes of the glycerate pathway in one operon: *gcl* (encoding glyoxylate carboligase), *hyi2* (hydroxypyruvate isomerase), *tsr* (tartronate semialdehyde reductase), and *ttuD1* (glycerate kinase). The latter enzyme probably generates 2-phosphoglycerate, which can be converted to 3PG via the glycolytic enzyme phosphoglycerate mutase (Pgam1,2). *C. necator* also harbors the key components of the C_2_ cycle: that is, the glycine cleavage system (encoded by *gcvTHP*) and serine hydroxymethyltransferase (*glyA*). While no dedicated glycine-dependent or serine-dependent transaminases are annotated in the genome of *C. necator* (Fig. 1), it is possible that glyoxylate amination and serine deamination are supported by one or more of the endogenous transaminase enzymes.

Finally, the *Synechocystis*-like oxalate decarboxylation pathway seems not to be present in *C. necator*, as no oxalate decarboxylase gene could be found. However, a similar route might be used. Specifically, oxalyl-CoA decarboxylase and formyl-CoA transferase—the genes for which, *oxc* and *frc*, are annotated and found in a single operon—could enable a glyoxylate oxidation route. In this putative route, glyoxylate is oxidized to oxalyl-CoA, which is then decarboxylated to formyl-CoA, converted to formate, and finally oxidized to CO_2_ (Fig. 1). However, no CoA-acylating glyoxylate dehydrogenase (catalyzing the first reaction of the pathway) is annotated in the genome of *C. necator*. Hence, we used Basic Local Alignment Search Tool (BLAST) to search the bacterium’s genome using the amino acid sequence of a CoA-acylating glyoxylate dehydrogenase from *Methylobacterium extorquens* (*panE2*) (28) as a query. We identified the gene *apbA2*, located in close proximity to the *frc-oxc* operon and showing high sequence homology to the *M. extorquens* protein (66% similarity, 51% identity), as a probable candidate CoA-acylating glyoxylate dehydrogenase.

To test whether ApbA2 can indeed catalyze the reversible CoA-acylating glyoxylate dehydrogenase reaction in vivo, we tested the growth of a Δ*apbA2* strain on oxalate. Growth on oxalate can proceed via two routes (*SI Appendix*, Fig. S1): 1) assimilation to central metabolism via the glycerate pathway, which depends on the activity of a CoA-acylating glyoxylate dehydrogenase (in the reverse direction of that required for growth on glycolate), and 2) complete oxidation of oxalate, which can be followed by carbon fixation via the Calvin cycle. Hence, if ApbA2 serves as a CoA-acylating glyoxylate dehydrogenase, its deletion could impair growth on oxalate. Indeed, while a wild-type *C. necator* grew efficiently on oxalate (doubling time of 6.5 ± 0.1 h) (*SI Appendix*, Fig. S1), growth of the Δ*apbA2* strain on this carbon source was impaired (doubling time of 10 ± 1.1 h) (*SI Appendix*, Fig. S1). This deletion strain showed a similar growth phenotype to that observed upon deletion of the enzymes of the glycerate pathway (doubling time of 13 ± 0.7 h) (*SI Appendix*, Fig. S1). It therefore seems that ApbA2 can indeed act as a CoA-acylating glyoxylate dehydrogenase. Interestingly, deletion of the *frc*-*oxc* operon completely abolished growth on oxalate (*SI Appendix*, Fig. S1), presumably as the supply of reducing power via oxalate oxidation is necessary to support the assimilation of this highly oxidized substrate.

By generating three distinct mutant strains carrying deletions in the *gcl*-*hyi2*-*tsr* operon, the *gcvTHP* operon, or the *frc-oxc* operon, we explored the contribution of each candidate route to growth on glycolate. While the deletion of the latter two operons did not affect growth on glycolate (doubling time of 3.4 ± 0.1 h) (ΔC2 ΔOX ΔMC in Fig. 2*A*), deletion of the *gcl*-*hyi2*-*tsr* operon resulted in a substantially lower growth rate (doubling time of 7.9 ± 0.1 h) (ΔGP in Fig. 2*A*), indicating that the glycerate pathway is the main route for growth on this carbon source (Fig. 2*B*). Still, the ability of the strain lacking the *gcl*-*hyi2*-*tsr* operon to grow on glycolate implies that other routes can support glyoxylate metabolism.

Next, we deleted the *gcvTHP* operon or the *frc-oxc* operon in the strain already lacking the *gcl*-*hyi2*-*tsr* operon. We found that further disruption of the oxalyl-CoA decarboxylation pathway substantially reduced the growth rate (doubling time of 22 ± 0.1 h) (ΔGP ΔOX in Fig. 2*A*), indicating that this route supports glyoxylate metabolism in the absence of the glycerate pathway. On the other hand, deletion of *gcvTHP*, blocking the C_2_ pathway, had only a small negative effect on growth (doubling time of 9.6 ± 0.1 h) (ΔGP ΔC2 in Fig. 2*A*), suggesting that this route contributes only marginally to the metabolism of glyoxylate.

Even after deleting all three operons, growth on glycolate was still observed (doubling time of 27 ± 0.1 h) (ΔGP ΔC2 ΔOX in Fig. 2*A*), indicating that additional unknown route(s) can support glyoxylate metabolism. Nevertheless, as analyzed comprehensively in *SI Appendix*, *Supplementary Text*, only a few other enzymes and pathways can potentially recycle glyoxylate, and even fewer of these are found in *C. necator*. For example, the β-hydroxyaspartate cycle was recently shown to enable growth on glycolate via glyoxylate assimilation (29), but the genome of *C. necator* does not encode its key glyoxylate assimilating enzyme, β-hydroxyaspartate aldolase. Of the very few metabolic pathways that might be involved in glyoxylate metabolism in *C. necator* (*SI Appendix*, *Supplementary Text*), the most plausible route relies on the enzyme malate synthase, a key component of the glyoxylate shunt that condenses glyoxylate with acetyl-CoA to generate malate (30, 31). Indeed, when the gene encoding malate synthase (*aceB*) was deleted in the strain lacking the *gcl*-*hyi2*-*tsr* operon, growth on glycolate was completely abolished (ΔGP ΔMC in Fig. 2*A*). This suggests that the activity of the oxalyl-CoA decarboxylation route on its own is too low to enable growth (e.g., due to low expression levels or poor enzyme kinetics). Notably, deletion of *aceB* in a strain in which the glycerate pathway is still active did not affect growth on glycolate (ΔC2 ΔOX ΔMC in Fig. 2*A*), emphasizing that the glycerate pathway is the main route supporting growth on this carbon source.

For glyoxylate metabolism via malate synthase to proceed, the cosubstrate acetyl-CoA must be regenerated. Such regeneration requires malate to undergo oxidative decarboxylation twice, first to pyruvate and then to acetyl-CoA. The combined activity of the malic enzyme and pyruvate dehydrogenase can support this double oxidation. The result is a “malate cycle,” composed of malate synthase, malic enzyme, and pyruvate dehydrogenase, which together completely oxidize glyoxylate to CO_2_ while generating two NAD(P)H molecules (Fig. 1). (We note that this cycle could alternatively proceed via malate oxidation to oxaloacetate, which is then converted to phosphoenolpyruvate via phosphoenolpyruvate carboxykinase and further metabolized to pyruvate and acetyl-CoA; this alternative malate cycle would result in the same net decarboxylation reaction.)

*C. necator* is expected to grow on glycolate via the malate cycle only by using the generated reducing power to fix CO_2_ via the Calvin cycle (Fig. 2*C*). To test if this is indeed the case, we deleted all genes encoding for Rubisco (*cbbS2*, *cbbL2*, *cbbSp*, and *cbbLp*) in the strain deleted in the *gcl*-*hyi2*-*tsr* operon. As anticipated, this strain was not able to grow on glycolate (ΔGP ΔRub in Fig. 2*A*); note that the deletion of Rubisco in a wild-type strain did not affect growth on glycolate as the glycerate pathway is still active (doubling time of 3.3 h) (ΔRub in Fig. 2*A*). These results confirm that growth on glycolate via the malate cycle strictly depends on carbon fixation via the Calvin cycle.

To summarize, growth on glycolate mainly depends on the glycerate pathway in a wild-type *C. necator* (Fig. 2*A*). When this route is disrupted, glyoxylate is metabolized via a combination of the malate cycle and the oxalyl-CoA decarboxylation pathway, both of which depend on CO_2_ fixation for growth (Fig. 2*C*). Under these conditions, the malate cycle carries most of the flux and is essential for growth, while the oxalyl-CoA decarboxylation pathway has a secondary role. Yet, it is still not clear whether the pathways that support growth on glycolate also participate in phosphoglycolate salvage during autotrophic growth at ambient CO_2_.

### Phosphoglycolate Salvage in *C. necator* Is Supported by the Glycerate Pathway and the Malate Cycle.

**Fig. 3.**
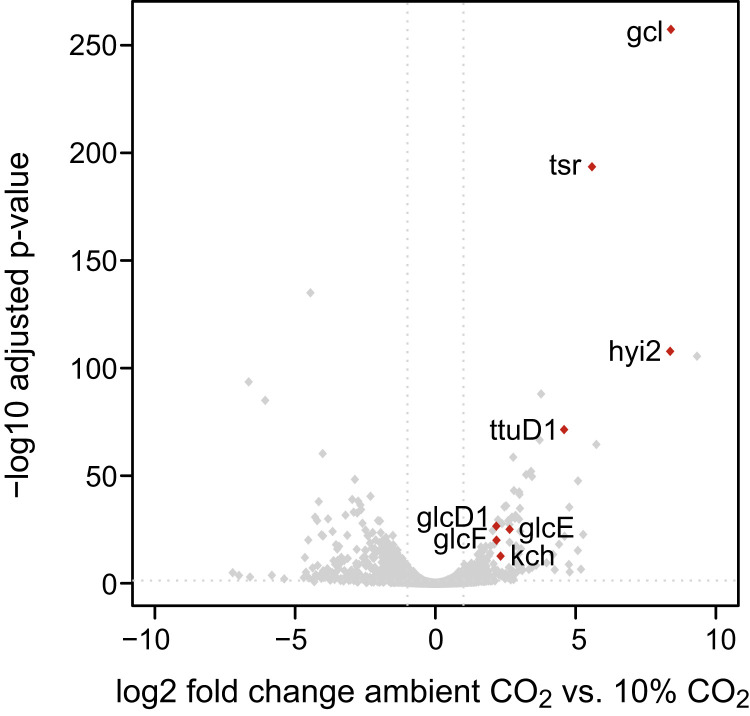
Volcano plot comparing the *C. necator* transcriptome for autotrophic growth at ambient CO_2_ vs. 10% CO_2_. RNA was isolated during midlog phase from *C. necator* grown in shake-flask cultures on minimal medium (JMM) with a headspace consisting of ambient air and 4% hydrogen or 10% CO_2_ and 4% hydrogen. Differential expression analysis was performed as described in *Materials and Methods*; log2-transformed fold changes and adjusted *P* values of all genes are depicted in the graph. Only genes that are significantly up-regulated and related to phosphoglycolate salvage pathways are labeled in the plot (red dots). Values for all genes can be found in Dataset S1. *gcl*, glyoxylate carboligase; *glcD1, glcE, glcF*, glycolate dehydrogenase complex subunits; *hyi2*, hydroxypyruvate isomerase; *kch*, putative glycolate dehydrogenase subunit; *tsr*, tartronate semialdehyde reductase; *ttuD1*, glycerate kinase.

To study which of the pathways of glycolate metabolism also participates in phosphoglycolate salvage, we compared the transcript levels of a wild-type *C. necator* growing autotrophically on hydrogen at ambient CO_2_ concentrations (∼0.04%) vs. elevated CO_2_ concentrations (10%) that suppress the oxygenation reaction. We found that the genes encoding for the Calvin cycle enzymes were overexpressed between 4- and 12-fold under ambient CO_2_ concentration (Dataset S1). This result is expected as higher activity of the cycle is needed to compensate for the decreased rate of Rubisco as well as the carbon loss from the oxygenation reaction and subsequent phosphoglycolate salvage. Furthermore, the genes encoding the first steps of phosphoglycolate salvage (that is, 2PG phosphatase [*cbbZ2* and *cbbZp*] and the glyoxylate dehydrogenase complex) were overexpressed between four- and eightfold (Fig. 3 and Dataset S1).

Genes encoding the enzymes of the glycerate pathway were among the most highly up-regulated at ambient CO_2_ (Fig. 3 and Dataset S1): *gcl* and *hyi2* were more than 300-fold overexpressed, *tsr* was ∼50-fold up-regulated, and *ttuD1* was ∼25-fold up-regulated. On the other hand, the genes related to the other candidate pathways for glyoxylate metabolism—that is, the C_2_ pathway, the oxalyl-CoA decarboxylation pathway, and the malate cycle—were not overexpressed at ambient CO_2_ (Dataset S1). This suggests that the glycerate pathway is the main route for phosphoglycolate salvage. However, the fact that the genes of the other pathways were not overexpressed does not necessarily mean that they do not participate in phosphoglycolate salvage. Specifically, it could be that their basal expression levels are sufficient to support the required activity. For example, the genes encoding for the components of the malate cycle—*aceB*, *maeA*, *maeB*, *pdhA1*, *pdhB*, and *pdhL*—are highly expressed under both ambient and 10% CO_2_ concentrations (all among the 10% most highly expressed in both conditions) (Dataset S1); hence, the malate cycle could play a role in phosphoglycolate salvage.

**Fig. 4.**
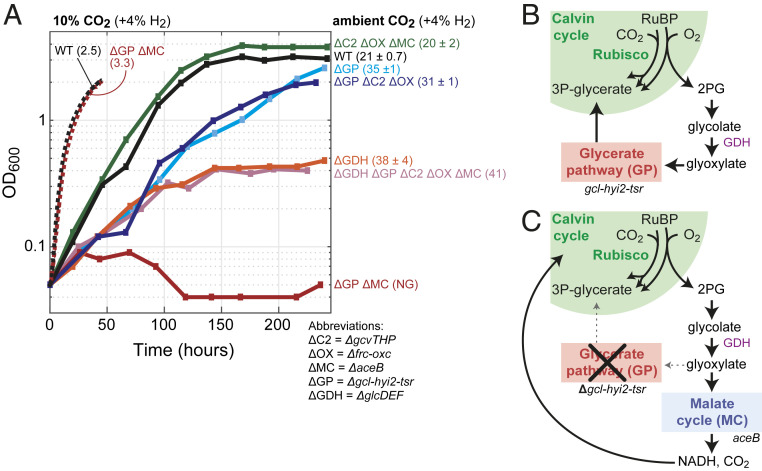
Autotrophic growth of *C. necator* gene deletion strains under ambient CO_2_. (*A*) Growth experiments were conducted in 700-mL bioreactor cultures on minimal medium (JMM) with a continuous sparging of gas (6.25 L/min) with ambient air + 4% hydrogen; control experiments were conducted at 10% CO_2_ + 4% hydrogen (supplemented with air). Doubling times (hours) and SDs are shown in parentheses; NG corresponds to no growth. Growth experiments on ambient CO_2_ were performed in biological duplicates and showed identical growth curves (±5%); hence, representative curves of a single experiment are shown. We performed control growth experiments on high CO_2_ for two strains only once and only up to 50 h to limit extremely high CO_2_ turnover in the (nonrecycling) bioreactor setup. (*B*) Phosphoglycolate salvage in *C. necator* proceeds mainly via glycolate dehydrogenase and the glycerate pathway to regenerate 3PG for the Calvin cycle. (*C*) In the absence of the glycerate pathway, glyoxylate is decarboxylated by the malate cycle to CO_2_ and NADH, which can be reassimilated by the Calvin cycle. ΔC2, C_2_ cycle knockout (*ΔgcvTHP*); ΔGDH, glycolate dehydrogenase knockout (*ΔglcD-kch-glcE-glcF*); ΔGP, glycerate pathway knockout (*Δgcl-hyi-tsr*); ΔMC, malate cycle knockout (*ΔaceB*); ΔOX, oxalate decarboxylation knockout (*Δfrc-oxc*); WT, wild type.

To determine the relative importance of each candidate pathway in phosphoglycolate salvage, we tested wild-type *C. necator* and several of the gene deletion strains described above for their ability to grow autotrophically at ambient CO_2_ (Fig. 4). Autotrophic growth of wild-type *C. necator* under these conditions resulted in a much lower growth rate than observed at 10% CO_2_ (doubling time of 21 ± 0.7 vs. ∼3 h) (solid vs. dashed WT lines in Fig. 4, respectively). This difference is expected from the lower carboxylation rate of Rubisco at low CO_2_ concentrations and the relatively high rate of the oxygenation reaction, which leads to CO_2_ release, thus directly counteracting carbon fixation.

A strain lacking all routes of glyoxylate metabolism other than the glycerate pathway did not show reduced growth at ambient CO_2_ (doubling time of 20 ± 2 h) (ΔC2 ΔOX ΔMC in Fig. 4). On the other hand, a strain in which the glycerate pathway was deleted displayed a substantially lower growth rate (doubling time of 35 ± 1 h) (ΔGP in Fig. 4). This suggests that the glycerate pathway is the major route of phosphoglycolate salvage but also, that it can be replaced by other pathways. Further deletion of the malate cycle in the strain lacking the glycerate pathway completely abolished autotrophic growth at ambient CO_2_ (ΔGP ΔMC in Fig. 4). On the other hand, disruption of the C_2_ pathway and the oxalyl-CoA decarboxylation pathway in the strain lacking the glycerate pathway did not alter its growth phenotype (doubling time of 31 ± 1 h) (ΔGP ΔC2 ΔOX in Fig. 4). These results clearly show that the malate cycle can participate in phosphoglycolate salvage, while the other two pathways contribute little, if any, to this process. Notably, deletion of both the glycerate pathway and the malate cycle did not affect autotrophic growth at high CO_2_ concentrations (dashed ΔGP ΔMC line in Fig. 4), as phosphoglycolate salvage is expected to be negligible under these conditions due to competitive inhibition of oxygenation at high CO_2_.

**Fig. 5.**
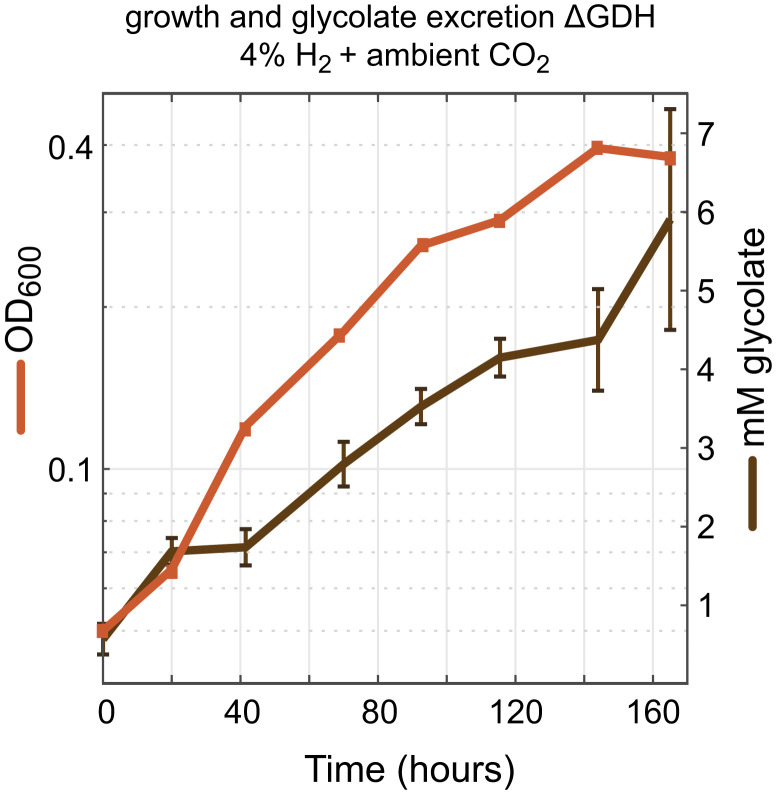
Glycolate excretion during autotrophic growth of the glycolate dehydrogenase knockout strain. Growth (OD_600_) and glycolate concentrations were monitored every 24 h during the growth phase of a *C. necator ΔglcD-kch-glcE-glcF* strain. Growth experiments were conducted in 700-mL bioreactor cultures on minimal medium (JMM) with a continuous sparging of gas (6.25 L/min) with ambient air + 4% hydrogen. Biological duplicates were measured, and SDs for the glycolate concentrations are shown; replicates showed identical growth curves (±5%), and a representative curve is shown. Glycolate was measured by ion chromatography as explained in *Materials and Methods*.

We were also interested to explore the outcome of disrupting phosphoglycolate salvage upstream of glyoxylate, following previous reports of glycolate secretion into the medium (24, 25). We found that a strain deleted in glycolate dehydrogenase could grow autotrophically under ambient CO_2_ concentration, albeit at substantially lower growth rate and yield (doubling time of 38 ± 4 h) (ΔGDH in Fig. 4). Supporting previous studies (24, 25), we found that glycolate accumulates in the medium during autotrophic growth of this strain at ambient CO_2_ (Fig. 5). No glycolate was detected during the autotrophic growth of a wild-type strain under the same conditions. Since glycolate is secreted by the strain lacking glycolate dehydrogenase and is not further oxidized, we hypothesized that further deletion of all four glyoxylate metabolism routes would not affect the growth phenotype; we indeed found this to be the case (doubling time of 41 h) (ΔGDH ΔGP ΔC2 ΔOX ΔMC in Fig. 4).

It therefore seems that *C. necator* can excrete glycolate if necessary, thus enabling the autotrophic growth of a strain lacking glycolate dehydrogenase. In contrast, *C. necator* seems to be incapable of secreting glyoxylate, which prevents the growth of a strain lacking the glycerate pathway and the malate cycle. Indeed, we could not detect glyoxylate in the growth medium of any of the tested strains.

### The Malate Cycle Might Be Prevalent in Chemolithoautotrophs Using the Calvin Cycle.

To explore the potential distribution of the malate cycle, we searched for the occurrence of malate synthase (PFAM 01274) (32) in bacteria that harbor a bona fide Rubisco and are therefore likely to operate the Calvin cycle and rely on phosphoglycolate salvage (*Materials and Methods*). Only 2% of cyanobacterial species harbor malate synthase, indicating that the malate cycle is likely uncommon among oxygenic photoautotrophic bacteria (Table 1). However, of the remaining ∼1,900 noncyanobacterial genomes found to encode Rubisco, ∼60% also encode a malate synthase. These include bacteria that grow chemolithoautotrophically by oxidizing either inorganic compounds (e.g., hydrogen, ammonia, and sulfur compounds) or organic one-carbon compounds (e.g., formate or methanol), as well as nonoxygenic phototrophs that use the Calvin cycle (such as purple nonsulfur bacteria). In several key orders of aerobic autotrophic bacteria, most genomes that harbor a Rubisco also encode a malate synthase (Table 1), including *Burkholderiales* (73%), *Chromatiales* (70%), *Rhizobiales* (98%), *Rhodobacterales* (88%), *Thiotrichales* (100%), *Mycobacteriales* (98%), and *Sulfobacilliales* (71%).

**Table 1.** Prevalence of malate synthase in chemolithoautotrophs and cyanobacteria that use the Calvin cycle

Phylum and order	Genera examples	Type of organisms	Electron donor	With Rubisco, no.	With malate synthase, %
Oxygenic photoautotrophs				**536**	**2**
Cyanobacteria		Photoautotroph	Water (oxygenic photosynthesis)	536	2
Chemolithoautotrophs				**1,890**	**63**
Proteobacteria					
Acidithiobacillales	*Acidithiobacillus*	Chemolithoautotrophs	Iron, hydrogen, sulfur compounds	9	0
Burkholderiales	*Cupriavidus*, *Nitrosomonas*	Chemolithoautrophs,	Hydrogen, formate, carbon monooxide, ammonia, sulfur compounds, iron	405	73
Chromatiales	*Allochromatium*, *Thiocapsa*	Chemolithoautotrophs, purple sulfur bacteria (anoxygenic phototrophs)	Hydrogen, sulfur compounds	40	70
Ectothiorhodospirales	*Thioalkalivibrio*	Chemolithoautotrophs, purple sulfur bacteria (anoxygenic phototrophs)	Sulfur compounds	35	40
Halothiobacillales	*Nitrosococcus*	Chemolithoautotrophs	Sulfur compounds	6	0
Nitrosococcales	*Halothiobacillus*	Chemolithoautotrophs	Ammonia	14	0
Rhizobiales	*Nitrobacter*, *Rhodopseudmonas*	Chemolithoautrophs, purple nonsulfur bacteria (anoxygenic phototrophs), methylotrophs	Hydrogen, nitrite, methanol, formate	305	98
Rhodobacterales	*Rhodobacter*, *Paracoccus*	Chemolithoautrophs, purple nonsulfur bacteria (anoxygenic phototrophs), methylotrophs	Hydrogen, sulfur compounds, methanol	150	88
Rhodospirillales	*Rhodospirillum*	Chemolithoautrophs, purple nonsulfur bacteria (anoxygenic phototrophs)	Hydrogen	46	39
Thiomicrospirales	*Hydrogenvibrio*	Chemolithoautrophs	Hydrogen, sulfur compounds	39	15
Thiotrichales	*Thiotrix*	Chemolithoautrophs	Sulfur compounds	7	100
Actinobacteriota					
Mycobacteriales	*Mycobacterium*	Methylotrophs, carboxidotrophs	Carbon monooxide, methanol, hydrogen	133	98
Firmicutes					
Sulfobacillales	*Sulfobacillus*	Chemolithoautotrophs	Sulfur compounds	7	71

The table shows only orders that include chemolithoautotrophs that are able to grow aerobically. Dataset S2 shows all orders and species and further shows the presence of isocitrate lyase. Numbers in bold indicate total numbers of oxygenic photoautotrophs and chemolithoautotrophs in all orders in these categories.

The presence of malate synthase does not necessarily indicate that the malate cycle plays a role in phosphoglycolate salvage. As mentioned above, the usual function of malate synthase is to support growth on acetate via the glyoxylate shunt, where it operates in concert with isocitrate lyase. Hence, we explored the co-occurrence of malate synthase and isocitrate lyase in bacteria harboring a Rubisco gene. As expected, in most cases (∼80%), bacteria with a malate synthase gene also encode an isocitrate lyase. Still, it should be noted that many bacteria have the capacity for both heterotrophic growth on acetate and autotrophic growth. Hence, even if the primary function of malate synthase is to support growth on acetate, it could still play a key role in phosphoglycolate salvage, as is the case in *C. necator*. The few notable exceptions, which were found to have a malate synthase without an isocitrate lyase, include bacteria of the orders *Rhodobacterales*, *Rhodospirillales*, and *Sulfobacillales* (Dataset S2). In these bacteria, the primary role of malate synthase might indeed be phosphoglycolate salvage.

The other enzymes of the malate cycle—the malic enzyme and pyruvate dehydrogenase or their alternatives (e.g., malate dehydrogenase, phosphoenolpyruvate carboxykinase)—are quite ubiquitous. However, these enzymes are not strictly necessary. While growth on glycolate via the malate cycle must be accompanied by the regeneration of acetyl-CoA and thus, complete oxidation of glyoxylate, in phosphoglycolate salvage acetyl-CoA does not need to be regenerated. Rather, the glyoxylate produced in phosphoglycolate salvage can be condensed with acetyl-CoA generated from the carbon fixation process, and the resulting malate is assimilated into biomass (i.e., via the tricarboxylic acid [TCA] cycle, cataplerosis, and gluconeogenesis). In this case, the malate “cycle” is not a real cycle but rather, represents a linear phosphoglycolate salvage route to reassimilate glyoxylate into central metabolism. We strongly suspect that some chemolithoautotrophs using malate synthase as part of their phosphoglycolate salvage actually operate the linear rather than the cyclic version of this pathway.

## Discussion

This study aimed to fill gaps in our knowledge of phosphoglycolate salvage in chemolithoautotrophic microorganisms. In *C. necator*, we confirmed the role of the glycolate dehydrogenase complex in phosphoglycolate salvage and further revealed two metabolic routes that can sustain glyoxylate assimilation during phosphoglycolate salvage at ambient CO_2_.

The glycolate dehydrogenase complex of *C. necator* is homologous to other bacterial glycolate dehydrogenases. The cofactor used as an electron acceptor for glycolate oxidation has not yet been elucidated in any bacteria. It is sometimes proposed, especially in cyanobacterial phosphoglycolate salvage, that NAD^+^ serves as the electron acceptor. However, this is highly doubtful as the change in Gibbs energy for the reaction glycolate + NAD^+^ = glyoxylate + NADH is very high (Δ_r_G′^m^ > 40 kJ/mol, pH 7.5, ionic strength of 0.25 mM; Δ_r_G′^m^ corresponds to metabolite concentration of 1 mM) (33). Instead, it is more likely that glycolate transfers its electrons, via a flavin adenine dinucleotide cofactor, to a quinone, the reduction potential of which is substantially higher than that of NAD (E′^m^ ∼ 0 mV rather than ∼−300 mV, respectively).

In addition to autotrophic growth, *C. necator* can also grow heterotrophically on various organic compounds, including glycolate. We used this metabolic versatility to explore phosphoglycolate salvage via a proxy process of glycolate metabolism. Indeed, we found that phosphoglycolate salvage and growth on glycolate mainly rely on the same routes: that is, the glycerate pathway and the malate cycle. In contrast, the oxalyl-CoA decarboxylation pathway seems to participate only in glycolate metabolism but not in phosphoglycolate salvage. This might be explained by the higher intracellular glyoxylate concentrations expected when feeding with external glycolate, which could induce the expression of the oxalyl-CoA decarboxylation pathway. Furthermore, the higher concentrations of glyoxylate could substantially increase the flux via this route if the affinity of its first enzyme, CoA-acylating glyoxylate dehydrogenase, toward glyoxylate is low. Further studies, analyzing the expression profile of the oxalyl-CoA decarboxylation pathway and the kinetics of its enzymes, could explain its role in glycolate metabolism and its absence in phosphoglycolate salvage.

Autotrophic growth phenotypes of our *C. necator* gene deletion strains show that the C_2_ pathway is a negligible contributor to phosphoglycolate salvage and glycolate metabolism in this bacterium. While *C. necator* harbors the main components of this pathway, transaminase enzymes that accept glycine and serine could not be identified. Although *C. necator* harbors multiple transaminases, it is virtually impossible to predict the precise substrate specificities of these highly promiscuous enzymes from their sequence. Therefore, it could be that *C. necator* simply lacks transaminases that can accept glyoxylate and serine and hence, cannot operate the C_2_ pathway. Alternatively, it could be that endogenous transaminases can aminate glyoxylate and deaminate serine, but the C_2_ pathway shows only low activity due to inadequate regulation.

A key finding of our work is the existence of the malate cycle as a supporting route for phosphoglycolate salvage and growth on glycolate. It is difficult to determine whether this route plays a role in the wild-type strain, as its deletion does not seem to hamper growth when the glycerate pathway is present. It could be that the malate cycle carries nonnegligible flux only when the glycerate pathway is disrupted and glyoxylate begins to accumulate. Alternatively, considering the relative high expression of the genes of the malate cycle, it is possible that the pathway always supports a substantial fraction of glyoxylate metabolism but not enough to affect growth when deleted.

The glycerate pathway is the most efficient naturally occurring phosphoglycolate salvage route in terms of consumption of adenosine triphosphate (ATP) and reducing power (34). The C_2_ pathway is less efficient, mainly due to the release of ammonia that needs to be reassimilated in a process that usually consumes ATP (at least in plants). The complete decarboxylation of glyoxylate to CO_2_—as supported by the cyanobacterial oxalate decarboxylation pathway as well as the oxalyl-CoA decarboxylation pathway and the malate cycle described here—is arguably the least efficient phosphoglycolate salvage mode, as it requires higher activity of the Calvin cycle to compensate for the lost carbon. This might explain why the deletion of the glycerate pathway in *C. necator*, such that the malate cycle carries phosphoglycolate salvage on its own, resulted in lower growth rate and yield (Fig. 4). The superiority of the glycerate pathway might also explain why it serves as the major phosphoglycolate salvage route both in cyanobacteria and in *C. necator*.

Despite the relative inefficiency of the malate cycle, its implementation in plants was suggested to boost carbon fixation and photosynthesis (34–37). Recently, the heterologous expression of malate synthase and glycolate dehydrogenase within the chloroplast of the agricultural crop *Nicotiana tabacum* led to a substantial increase in photosynthetic rate and yield (35). As the malate cycle is less efficient than the natural C_2_ pathway, this growth enhancement is not easy to explain and was suggested to be related to the release of CO_2_ in the chloroplast, rather than the mitochondria, thus suppressing the oxygenase side reaction and promoting Rubisco’s carboxylation.

As malate synthase is present in many chemolithoautotrophs that use the Calvin cycle (Table 1), it is tempting to suggest that it contributes to phosphoglycolate salvage in many of these bacteria. However, the occurrence of malate synthase in cyanobacteria is much lower (Table 1). *Synechocystis*, the only bacterial photoautotroph for which phosphoglycolate salvage has been physiologically characterized (5, 8), probably lacks malate synthase and hence, cannot operate the malate cycle (38, 39). However, the presence of malate synthase has been confirmed in some cyanobacteria, leading to the suggestion that they may use the malate cycle (40–43). We leave it for future studies to explore the occurrence of the malate cycle and other phosphoglycolate salvage routes in cyanobacteria and chemolithoautotrophs. Such investigations will generate further insights into the evolutionary history of phosphoglycolate salvage and carbon fixation via the Calvin cycle.

## Materials and Methods

### Strains, Conjugations, and Gene Deletions.

*C. necator* H16 (Deutsche Sammlung von Mikroorganismen und Zellkulturen [DSMZ] 428) was used for transcriptome studies. Growth experiments were performed for a *C. necator* H16 strain knocked out for polyhydroxybutyrate biosynthesis (*ΔphaC1*) (44), in which other gene deletions were performed. The *ΔphaC1* strain grows in nutrient nonlimiting conditions similar to the wild type and does not result in polyhydroxybutyrate (PHB) granules that could disturb optical density measurements.

Cloning of plasmids was performed in *Escherichia coli* DH5α, whereas *E. coli* S17-1 was used for conjugation of mobilizable plasmids to *C. necator* by biparental overnight spot mating. A complete overview of strain genotypes used in this study can be found in Table 2.

**Table 2.** Strains used in this study

Strain	Description	Source
*C. necator* H16	*C. necator* H16 (DSMZ 428)	DSMZ
Wild type	*C. necator* H16 *ΔphaC1*	Ref. 44
ΔC2 ΔOX ΔMC	*C. necator* H16 *ΔphaC1*	This study
ΔΔRubisco	*C. necator* H16 *ΔphaC1 ΔcbbL2-cbbS2 ΔcbbLp-cbbSp*	This study
ΔGP	*C. necator* H16 *ΔphaC1 Δgcl-hyi2-tsr*	This study
ΔGP ΔC2	*C. necator* H16 *ΔphaC1 Δgcl-hyi2-tsr ΔgcvTHP*	This study
ΔGP ΔOX	*C. necator* H16 *ΔphaC1 Δfrc-oxc*	This study
ΔGP ΔC2 ΔOX	*C. necator* H16 *ΔphaC1 Δgcl-hyi2-tsr ΔgcvTHP Δfrc-oxc*	This study
ΔGP ΔMC	*C. necator* H16 *ΔphaC1 ΔaceB*	This study
ΔGP ΔRub	*C. necator* H16 *ΔphaC1 Δgcl-hyi2-tsr ΔcbbL2-cbbS2 ΔcbbLp-cbbSp*	This study
ΔGDH	*C. necator* H16 *ΔphaC1 ΔglcD-kch-glcE-gclF*	This study
ΔGDH ΔGP ΔC2 ΔOX ΔMC	*C. necator* H16 *ΔphaC1 ΔglcD-kch-glcE-gclF Δgcl-hyi2-tsr ΔgcvTHP Δfrc-oxc ΔaceB*	This study
*E. coli* S17-1	*recA pro thi-1 hsdR*	Ref. 45
	*RP42Tc::MuKm::Tn7* integrated into the chromosome (DSM 9079)	
*E. coli* DH5α	*fhuA2Δ(argF-lacZ)U169 phoA glnV44 Φ80Δ (lacZ)M15 gyrA96 recA1 relA1 endA1 thi-1 hsdR17E. coli* S17-1 *λpir ΔhemaA*	Ref. 46

Gene deletions were performed with the pLO3 suicide vector, as previously described (47, 48). Briefly, ∼1-kb homology arms upstream and downstream of the gene or operon to be deleted were amplified using PCR (Phusion HF polymerase with dimethyl sulfoxide [DMSO]; Thermo Scientific) from genomic *C. necator* DNA using primers as listed in *SI Appendix*, Table S1. Homology arms were cloned into pLO3 backbone (SacI, XbaI digested) via In-Fusion Assembly (Takara) and confirmed by Sanger Sequencing (LGC). *C. necator* was conjugated with *E. coli* S17-1 harboring pLO3 knockout vectors, and single-homologous recombination clones were selected on agar plates with tetracycline (10 µg/mL) and 10 µg/mL gentamycin to counterselect for *E. coli*. Next, some transconjugants were grown in an overnight liquid culture (without tetracycline) to support a second homologous recombination event. The overnight cultures were plated on Lysogeny Broth (LB) with 10% sucrose to allow for SacB counterselection. Resulting colonies were screened by colony PCR (OneTaq; Thermo Scientific) to identify gene-deleted strains (primers are in *SI Appendix*, Table S1), which were further verified for having lost tetracycline resistance. Biological duplicates of each knockout strain were constructed.

### Growth Medium and Conditions.

*C. necator* and *E. coli* were cultivated for routine cultivation and genetic modifications on LB (1% NaCl, 0.5% yeast extract, 1% tryptone). Routine cultivation was performed in 3 mL medium in 12-mL glass culture tubes in a Kuhner shaker incubator (240 rpm) at 30 °C for *C. necator* and 37 °C for *E. coli*. Growth characterization and transcriptomic experiments of *C. necator* were performed in J Minimal Medium (JMM) as reported previously (49).

### Ninety-Six–Well Plates Growth Experiments.

Growth on glycolate or oxalate was performed on JMM with 40 mM sodium glycolate or 40 mM sodium oxalate (Sigma-Aldrich). Experiments were performed in 96-well plates (Nunc transparent flat bottom; Thermo Scientific) under ambient air conditions. Precultures for these experiment were performed in JMM with 20 mM fructose in glass tubes, and next, they were washed three times and inoculated at an OD_600_ (optical density at 600 nm) of 0.01 or 0.005; 150 μL of culture medium was topped with 50 μL of transparent mineral oil (Sigma-Aldrich) to prevent evaporation (O_2_ and CO_2_ can diffuse well through this oil). Plates were incubated with continuous shaking, alternating between 1 min orbital and 1 min linear, in a BioTek Epoch 2 plate reader. OD_600_ values were recorded every ∼12 min. Growth data were processed by an in-house Matlab script, converting OD_600_ measured by the reader to cuvette OD_600_, by multiplication with 4.35. All growth experiments were performed at least in triplicates, and the growth curves shown are representative curves.

### Bioreactor Experiments.

Autotrophic growth was performed in bioreactor experiments. Strains were precultured in 100 mL JMM in 500-mL Erlenmeyer with 80 mM sodium formate and 10% CO_2_ in the headspace. Precultures were washed three times and resuspended in JMM without carbon source. Bioreactors (DASGIP; Eppendorf) with 700 mL JMM medium were inoculated at a starting OD_600_ of 0.05. The reactors were continuously sparged (6.25 L/min) with a gas mixture of 4% hydrogen and 96% ambient air (or 86% air + 10% CO_2_ for control experiments), controlled by a gas flow controller (HovaGas). The reactors were stirred at 200 rpm, and temperature was controlled at 30 °C. To compensate for high evaporation losses due to high sparging flow, a level sensor controlled a feed pump of sterile water. Samples for OD_600_ measurements and supernatant analysis were taken daily, while fast growth at 10% CO_2_ was monitored by an online sensor calibrated for cuvette OD_600_.

### RNA Isolation and Sequencing.

For transcriptome analysis, cells were grown in 10 mL JMM (without carbon source) in 100-mL Erlenmeyer within a 10-L desiccator filled with 4% hydrogen + 96% ambient air or 4% hydrogen + 10% CO_2_ and 86% air. To maintain hydrogen and CO_2_, the gas phase in the desiccator was exchanged at least twice a day. Biological replicate cultures were harvested in log phase (2 mL culture for OD_600_ ∼ 0.2 for ambient CO_2_, 1 mL for OD_600_ ∼ 0.4 for 10% CO_2_) and stabilized by the RNA Protect Bacteria Kit (Qiagen). Next, cells were lysed using lysozyme and a bead-beating step with glass beads (Retschmill; MM200) for 5 min at 30 Hz. RNA was purified using the RNeasy Mini kit (Qiagen) according to the manufacturer’s instructions and on-column DNase digestion (DNase kit; Qiagen). Ribosomal RNA (rRNA) depletion (RiboZero kit), complementary DNA (cDNA) library preparation, and paired-end 150-bp read sequencing (Illumina HiSeq 3000) were performed by the Max Planck Genome Centre Cologne, Germany.

### Transcriptome Data Analysis.

Sequence data of all samples were mapped with STAR v2.5.4b using default parameters (50). Ensembl version 38 genome reference in FASTA format and Ensembl version 38 cDNA Annotation in General Feature Format (GTF) were used for genome indexing with adapted parameters for genome size (–genomeSAindexNbases 10) and read length (–sjdbOverhang 150). Antistrand reads out of the ReadsPerGene files, which are automatically generated by STAR, were used in two different ways: calculating reads per kilobase of exon per million mapped reads for sample-wise transcript abundances as well as merging in order to perform a differential expression analysis with DESeq2 (51) as guided by the rnaseqGene Bioconductor workflow (https://bioconductor.org/packages/release/workflows/vignettes/rnaseqGene/inst/doc/rnaseqGene.html). Briefly, samples were grouped by single-parameter condition (ambient CO_2_ and 10% CO_2_), and read count data were then loaded with DESeqDataSetFromMatrix to create a DeSeqDataSet object to subsequently run the standard analysis consisting of the functions DESeq and Results. Then, log2-transformed fold changes for ambient CO_2_ compared with 10% CO_2_ and absolute log10 of adjusted *P* values were determined and were visualized in a volcano plot.

### Supernatant Analysis for Glycolate and Glyoxylate.

Glycolate concentrations in culture supernatant were determined by ion chromatography analysis. Supernatant samples were diluted 1:100 in ultrapure water (Milli-Q). The samples were analyzed in an ICS 3000 (Dionex) ion chromatography system, which was combined with an AS50 auto sampler. The samples were run through a Dionex IonPac AS11 IC column (4-mm diameter, 250-mm length [044076]) and a guard column (4-mm diameter, 50-mm length [044078]). Samples were run following KOH eluent gradient: 1 mM from 0 to 5 min, 1 to 15 mM from 5 to 14 min, 15 to 30 mM from 14 to 23 min, and 30 to 60 mM from 23 to 31 min at a flow rate of 0.015 mL/min. The experimental data were analyzed using Chromeleon 6.8. Concentrations were calculated based on a standard curve generated for sodium glycolate (Sigma-Aldrich) in JMM medium. Glyoxylate concentrations were determined by a colorimetric assay based on a reported protocol (52). Specifically, 216 µL from the supernatant sample were mixed with 24 µL of 1% wt/vol phenylhydrazine in 0.1 M HCl, incubated for 10 min at 60 °C, and cooled down. To 100 µL of this mixture, 50 µL concentrated HCl and 20 µL of 1.6% wt/vol potassium ferricyanide were added, while background control samples were prepared with 100 µL of reacted sample mixture with 50 µL concentrated HCl and 20 µL Milli-Q (MQ) water. These samples were incubated for exactly 12 min, and then, absorbance of 1,5-diphenylformazan at 520 nm was recorded by a BioTek Epoch 2. Differences in absorbance were calculated for each sample by subtracting absorbance from background controls, and glyoxylate concentration could be determined based on a standard curve. All supernatant samples of all autotrophic cultures in this work resulted in negligible levels of <0.1 mM glyoxylate.

### Genomic Prevalence Analysis of Rubisco and Malate Synthase.

Lists of bacterial genomes containing the large subunit of Rubisco (PF00016), malate synthase (PF01274), or isocitrate lyase (PF00463) were downloaded from the AnnoTree website (53) on 10 July 2020 by searching for the appropriate protein families. As of writing, AnnoTree uses version 89 of the Genome Taxonomy Database (GTDB) (54), which was retrieved from the GTDB website on the same date. Rubisco sequences were filtered by using usearch (55) to remove any amino acid sequences with >30% identity to known Rubisco-like proteins (type IV Rubiscos). The list of Rubisco-like proteins was drawn from ref. 56, and Rubisco-like proteins were removed because they do not catalyze the carboxylation reaction (57). Organisms encoding Rubisco and malate synthase or Rubisco, malate synthase, and isocitrate lyase were identified by using their GTDB identifications to merge the tables along with taxonomic information. This permitted calculation of the fraction of Rubisco-containing genomes that also contain malate synthase or malate synthase and isocitrate lyase for each order in the GTDB taxonomy. Analyses were performed in a custom Python script that is available in https://github.com/flamholz/malate_synthase/blob/master/pipeline/01_plot_co_occurrence.ipynb.

## Supplementary Material

Supplementary File

Supplementary File

Supplementary File

## Data Availability

Transcriptomic data are available in the Gene Expression Omnibus (accession no. GSE141999) (58) and are available in Dataset S1. Bioinformatic genome analysis of Rubisco and malate synthase are available in Dataset S2 and at GitHub (https://github.com/flamholz/malate_synthase/tree/master/pipeline) (59).
