# Supplementary material for: Phosphoglycolate salvage in a chemolithoautotroph using the Calvin cycle
Source: Proc Natl Acad Sci U S A. 2020 Aug 20;117(36):22452–61. doi: 10.1073/pnas.2012288117 (PMC7486775; doi:10.1073/pnas.2012288117)
Supplement: Supplementary File [file pnas.2012288117.sapp.pdf]

Supplementary Information belonging to "Phosphoglycolate salvage in a chemolithoautotroph using the Calvin cycle"

Nico J. Claassens†[1], Giovanni Scarinci†[1], Axel Fischer[1], Avi I. Flamholz[2], William Newell[1], Stefan Frielingsdorf[3], Oliver Lenz[3], Arren Bar-Even*[1]

Supplementary Text

Possible metabolic pathways supporting glycolate metabolism and phosphoglycolate salvage

In a previous study, we systematically analyzed possible metabolic routes that could assimilate phosphoglycolate and glycolate into central metabolism without releasing CO_2 (1). These candidate pathways do not involve oxidation reactions and hence do not have glyoxylate as an intermediate. Importantly, each of these pathways harbors at least one reaction that is only plausible, i.e., corresponds to an established enzymatic mechanism, but not known to be catalyzed by any natural enzyme. Hence, these non-oxidative routes are unlikely to exist in nature and, with high certainty, do not operate in *C. necator*.

Considering the initial oxidation of glycolate to glyoxylate, there are several possible routes by which the latter compound can be further metabolized:

1. **Glyoxylate oxidation**. In this case, the product would be either oxalate (EC 1.2.3.5) or oxalyl-CoA (EC 1.2.1.17). These compounds can then be decarboxylated and further oxidized to CO_2 (2, 3). While oxalate decarboxylase in missing in *C. necator*, the oxalyl-CoA route is present in this bacterium and contributes to glyoxylate metabolism. However, as discussed in the main text, the oxalyl-CoA route does not seem to participate in phosphoglycolate salvage in *C. necator*.

2. **Glyoxylate as an acceptor substrate for an aldolase reaction**. For example, 4-hydroxy-2-oxoglutarate aldolase (EC 4.1.3.16) condenses pyruvate (as a donor, i.e., nucleophile) and glyoxylate (as an acceptor, i.e., electrophile) to give 4-hydroxy-2-oxoglutarate. Various other aldolases (EC 4.1.3.-) can potentially use glyoxylate as an acceptor. However, many of these aldolases use a donor compound that is not readily available (not a central metabolism intermediate). Moreover, for all of the aldolases, the compounds resulting from the aldol condensation cannot be easily assimilated into central metabolism (e.g., 4-hydroxy-2-oxoglutarate) and the donor molecule cannot be easily recycled. Hence, such aldolase reactions are unlikely to contribute to glyoxylate metabolism and phosphoglycolate salvage in *C. necator*.

3. **Glyoxylate as an acceptor substrate for an oxo-acid-lyase reaction or an acyltransferase reaction**. A carboxylic acid or a thioester can serve as a donor (i.e., nucleophile) and glyoxylate as an acceptor (i.e., electrophile), where the thioester might be retained in the product (an oxo-acid-lyase reaction, EC 4.1.3.-) or hydrolyzed (an acyltransferase reaction, EC 2.3.3.-). Glyoxylate can serve as possible acceptor for many such enzymes but only few of them could lead to a compound that can be easily assimilated into central metabolism. These cases include:

   a. **Malate synthase / malyl-CoA lyase**. Condensation of acetyl-CoA with glyoxylate to generate malate – either directly (EC 2.3.3.9) or via a malyl-CoA intermediate (EC 4.1.3.24) – can be used to assimilate glyoxylate into central metabolism. If necessary, the donor acetyl-CoA could be recycled by malate oxidation, as described in the main text. While *C. necator* does not harbor a malyl-CoA lyase, it does have malate synthase. As discussed in details in the main text, this enzyme plays a key role in glyoxylate metabolism and phosphoglycolate salvage.

www.pnas.org/cgi/doi/10.1073/pnas.2012288117

b. **Methylmalate synthase / Methylmalyl-CoA lyase**. The enzymes described above can also condense propionyl-CoA with glyoxylate to give methylmalyl-CoA or methylmalate. Methylmalyl-CoA can be then converted to citramalyl-CoA, for example, by the enzymes of the 3-hydroxypropionate bicycle in *Chloroflexus aurantiacus* (4). Similarly, methylmalate can be converted to citramalate, for example, by the enzymes of the isoleucine biosynthesis pathway in *Leptospira interrogans* (5). Citramalyl-CoA or citramalate can then be cleaved to pyruvate and acetyl-CoA or acetate, thus entering central metabolism. The main problem with this route is that the donor propionyl-CoA is not part of central metabolism in most organisms. Specifically, in *C. necator*, which is not known to produce propionyl-CoA (e.g., via a succinyl-CoA decarboxylation route (6)) and also lacks the enzymes to convert methylmayl-CoA/methylmalate to citramalyl-CoA/citramalate, this pathway is highly unlikely to support glyoxylate metabolism or phosphoglycolate salvage.

c. **Isocitrate lyase**. Condensation of succinate with glyoxylate to generate isocitrate (EC 4.1.3.1) provides another route for glyoxylate assimilation to central metabolism. If necessary, the donor succinate can be recycled via oxidation of isocitrate, as occur naturally in the TCA cycle. As *C. necator* harbors all its necessary enzymes, this route could potentially participate in glyoxylate metabolism and phosphoglycolate salvage; however, no experimental evidence for its activity was found.

4. **Glyoxylate as an acceptor substrate for thiamine dependent condensation**. Thiamine dependent enzymes such as transketolase (EC 2.2.1.1), acetolactate synthase (EC 2.2.1.6), could condense an aldehyde moiety with glyoxylate (serving as an acceptor, i.e., electrophile). However, in most cases the resulting compound cannot be easily assimilated into central metabolism. For example, condensation of a glycolaldehyde moiety (derived from a phosphosugar) or an acetaldehyde moiety (derived from pyruvate) with glyoxylate would generate a C_4 metabolite far separated from central metabolism. The exception is tartronate semialdehyde synthase (EC 4.1.1.47), which uses glyoxylate both as donor and acceptor, effectively condensing a formaldehyde moiety with glyoxylate to generate tartronate semialdehyde, which can further be reduced to glycerate and assimilated to central metabolism. As discussed in the main text, these reactions provide the main rote for glyoxylate assimilation and phosphoglycolate salvage in *C. necator* as well as many other organisms.

5. **Amination of glyoxylate to glycine**. Glyoxylate can be aminated to glycine via the activity of a transaminase enzyme (EC 2.6.1.-) or a dehydrogenase enzyme (EC 1.4.1.10). The assimilation of glycine to central metabolism can proceed in three routes:

a. **Glycine to serine to glycerate**. As in plant photorespiration, glycine can be converted serine, which is then metabolized to hydroxypyruvate and glycerate (7). As discussed in the main text, this route seems not to take place in *C. necator*, since deletion of the glycine cleavage system does not hamper glyoxylate metabolism or phosphoglycolate salvage

b. **Glycine to serine to pyruvate**. Rather than being converted to glycerate, serine can be deaminated to pyruvate, as in the reductive glycine pathway (8). This pathway does not appear to operate in *C. necator*, since deletion of the glycine cleavage system does not hamper glyoxylate metabolism or phosphoglycolate salvage.

c. **Hydroxyaspartate pathway**. In the recently elucidated hydroxyaspartate cycle (9), glycine reacts with glyoxylate to generate hydroxyaspartate, which then undergoes dehydration and reduction to give aspartate, thus entering central metabolism. This route is a prime candidate to support glyoxylate assimilation and phosphoglycolate salvage, but seems to be absent in *C. necator* which does not harbor its enzymes.

Supplementary Figure

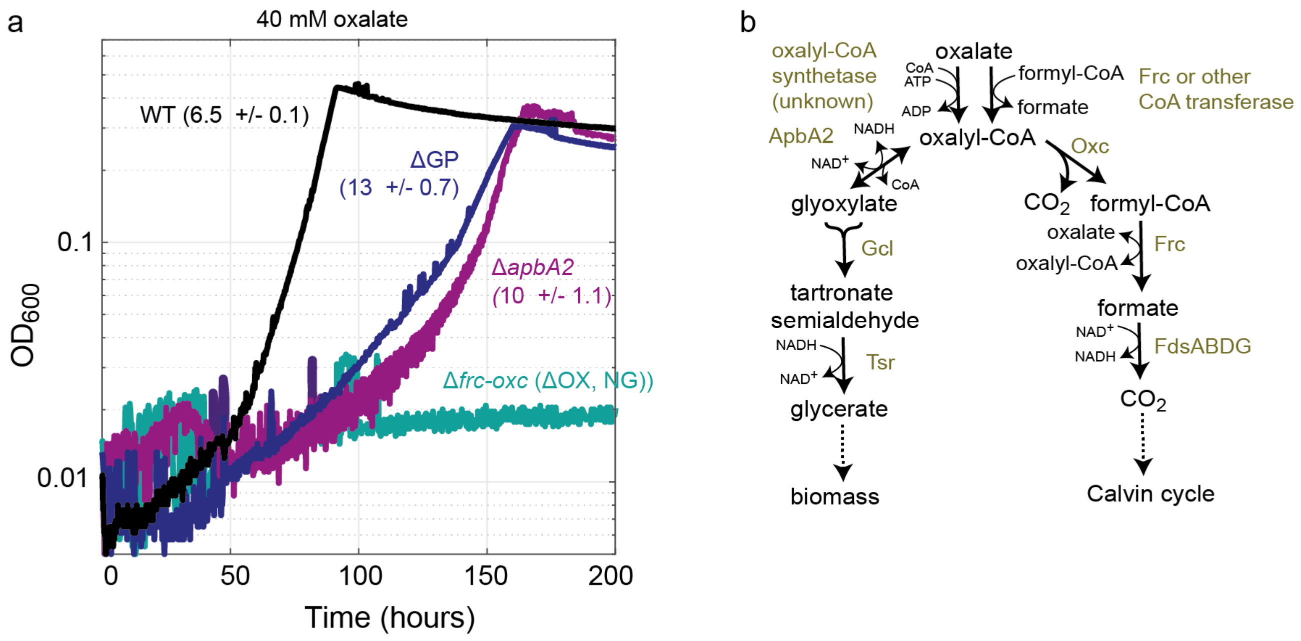

Supplementary Figure S1. Growth of *C. necator* on oxalate. (a) Growth experiment of gene-deletion strains on oxalate**.** Growth experiments were conducted in 96-well plate readers in minimal medium (JMM) supplemented with 40 mM sodium oxalate. Doubling times (hours) and standard deviations of triplicates are shown in between brackets, 'NG' corresponds to 'no growth'. Growth experiments were performed in biological triplicates and showed identical growth curves (±5%); hence, representative curves of single experiments are shown. **(b)** Growth on oxalate can proceed via two main routes in *C. necator*, the reduction of oxalyl-CoA to glyoxylate and its assimilation via the glycerate route, or via the oxidation of oxalyl-CoA and full decarboxylation to CO_2. Growth of gene-deleted strains for one of both routes show that both play a role in growth on oxalate, the decarboxylative route seems essential as its knockout abolished growth. Abbreviations: ApbA2, CoA-acylating glyoxylate dehydrogenase; FdsABDG, formate dehydrogenase complex; Frc, formyl-CoA transferase; Gcl, glyoxylate carboligase; ΔGP, glycerate pathway knockout (*Δgcl-hyi-tsr*); ΔOX, oxalate decarboxylation knockout (*frc-oxc*); Oxc, oxalyl-CoA decarboxylase; Tsr, tartronate semialdehyde reductase; TtuD1, glycerate kinase; and WT, wild-type.

Supplementary Table

Supplementary Table S1. Primers used in this study.

Gene/operon deletion	Gene ID(s)	Primer type	Primer sequence 5' – 3'
ΔgclD-kch-EF (ΔGDH)	H16_A3094-3097	Fw_HRup	cacctagatccttttaattcctggaggtggaatcggtct
		Rv_HRup	tgtcgaccagattcatgaacgactcctgtg
		Fw_HRdown	ccacctccagctggtcgacaagatgctcggcta
		Rv_HRdown	gtttaaacagtcgactctagttggtttgccatgttgatgt
		Fw_colPCR_1	gaggacggcactgacaagac
		Fw_colPCR_2	gacaagctcgacatggtctg
		Rv_colPCR	atcatgcctgctctcctttc
Δgcl-hyi2-tsr (ΔGP)	H16_A3598-3600	Fw_HRup	acctagatccttttaattcgtccatcggatattcgtctcc
		Rv_HRup	ggtggttagagctctcatctttgccatgatct
		Fw_HRdown	agatgagagctctaaccaccagatcgccaag
		Rv_HRdown	gtttaaacagtcgactctagattacgcagggcttgctg
		Fw_colPCR	actccaacggaattcacacc
		Rv_colPCR_1	cagcaggtcgatatgctcag
		Rv_colPCR_2	aaatcaggcacagcaccag
ΔgcvT1HP (ΔC2)	H16_A3619-3621	Fw_HRup	cacctagatccttttaattcggcaagggccgtccaggtag
		Rv_HRup	cgtagtcgctcatggaatcctctggggcagtc
		Fw_HRdown	ggattccatgagcgactacgtggtggactgag
		Rv_HRdown	gtttaaacagtcgactctaggttctcttcattgactgcgatgg
		Fw_colPCR	ggtatcgaggacagaggggtca
		Rv_colPCR	caggtcagctcgttttccat
frc-oxc (ΔOX)	H16_B1711-1712	Fw_HRup	acctagatccttttaattcgatagagctggcccaccgtagc
		Rv_HRup	agtcggaaacgagtcccaggtgatctgacgc
		Fw_HRdown	cctgggactcgtttccgacttctgccatgg
		Rv_HRdown	gtttaaacagtcgactctagttcctgtcggacaagttcgg
		Fw_colPCR	tctggtctgtcatggggatt
		Rv_colPCR	acttgaagggctaggggaaa
ΔaceB (ΔMC)	H16_A2217	Fw_HRup	acctagatccttttaattcgcgattaagcccgtaggcgacc
		Rv_HRup	aagactgatgctgacgctgccgctgtatgag
		Fw_HRdown	gcagcgtcagcatcagtcttctcctgtgatc
		Rv_HRdown	gtttaaacagtcgactctaggtctccagttgtttgaagtgg
		Fw_colPCR	ccctacccgcttaaacttcc
		Rv_colPCR	caggccctcatgaacaagat
ΔcbbLS2 (ΔRub)	H16_B1394-1395	Fw_HRup	acctagatccttttaattcgatggtggtcagggtatggtc
		Rv_HRup	ccaagccaagcatgcgctactcgatcgaga
		Fw_HRdown	gtagcgcatgcttggcttggaccgattcag
		Rv_HRdown	gtttaaacagtcgactctagacatatgcgcaacatgccagatg
		Fw_colPCR	atcaatcgagcatccgactc
		Rv_colPCR	acccctttgcatcgtgtaac
ΔcbbLSp (ΔRub)	PHG_426-427	Fw_HRup	acctagatccttttaattcgtttcgtcgtcaaagcggtac
		Rv_HRup	gacaagcatgtattcgatcgagagctacgcc
		Fw_HRdown	cgatcgaatacatgcttgtctccttgcgtg
		Rv_HRdown	gtttaaacagtcgactctagaatcccctaggccacaagcc
		Fw_colPCR	ggcttcagtccgatcagttc
		Rv_colPCR	aggaaggacatgagcagtgg
ΔapbA2	H16_B1719	Fw_HRup	acctagatccttttaattcgcaaaacggcggaccaaggtc
		Rv_HRup	gacagcaatgctgatcaagcagcgcgacac
		Fw_HRdown	gcttgatcagcattgctgtctccttgatgg
		Rv_HRdown	gtttaaacagtcgactctagagaactgtaagagacgccgc

Supplementary references

1. Trudeau DL*, et al.* (2018) Design and in vitro realization of carbon-conserving photorespiration. *Proc Natl Acad Sci U S A* 115(49):E11455-E11464.
2. Svedruzic D*, et al.* (2007) Investigating the roles of putative active site residues in the oxalate decarboxylase from Bacillus subtilis. *Arch Biochem Biophys* 464(1):36-47.
3. Werther T*, et al.* (2010) New insights into structure-function relationships of oxalyl CoA decarboxylase from Escherichia coli. *FEBS J* 277(12):2628-2640.
4. Zarzycki J, Brecht V, Muller M, & Fuchs G (2009) Identifying the missing steps of the autotrophic 3-hydroxypropionate CO2 fixation cycle in Chloroflexus aurantiacus. *Proc Natl Acad Sci U S A* 106(50):21317-21322.
5. Xu H*, et al.* (2004) Isoleucine biosynthesis in Leptospira interrogans serotype lai strain 56601 proceeds via a threonine-independent pathway. *J Bacteriol* 186(16):5400-5409.
6. Tholozan JL, Grivet JP, & Vallet C (1994) Metabolic pathway to propionate of Pectinatus frisingensis, a strictly anaerobic beer-spoilage bacterium. *Arch. Microbiol.* 162(6):401-408.
7. Fernie AR*, et al.* (2013) Perspectives on plant photorespiratory metabolism. *Plant Biol (Stuttg)* 15(4):748-753.
8. Kim S*, et al.* (2020) Growth of E. coli on formate and methanol via the reductive glycine pathway. *Nat Chem Biol* 16:538–545.
9. Schada von Borzyskowski L*, et al.* (2019) Marine Proteobacteria metabolize glycolate via the beta-hydroxyaspartate cycle. *Nature* 575(7783):500-504.
